# The effect of litter size and birth weight on the pre-weaning survivability of Kalahari Red goats

**DOI:** 10.1007/s11250-026-05062-1

**Published:** 2026-05-08

**Authors:** Tebogo Letsukulo Percy Thepa, Thobela Louis Tyasi, Jones Wilfred Ng’ambi

**Affiliations:** 1 Department of Agriculture and Animal Health, College of Agriculture and Environmental Sciences, University of South Africa, Florida, 1710 South Africa; 2 Department of Agricultural Economics and Animal Production, University of Limpopo, Private Bag X1106, Sovenga, Limpopo 0727 South Africa

**Keywords:** Goat breeding, Goat kids, Goat mortality, Reproductive performance, Survival rates

## Abstract

Pre-weaning mortality is a challenge for goat production systems, especially under extensive management. The study investigated the effect of the litter size and birth weight on the pre-weaning survivability of Kalahari Red goat kids. A longitudinal dataset which comprised of the litter size, birth weight and mortality records that occurred over 15 years (2009–2024) was retrieved from the database. A two-way analysis of variance was used for analysis. The results indicated that the litter size had a significant impact (*p* < 0.05) on the pre-weaning survivability of kids and pre-weaning survival rates were shown to decrease with an increasing litter size, from a survival rate of 95% in singletons to one of 69% in quintuplets. The results showed that the birth weight, nor its interaction with litter size, were not significantly (*p* > 0.05) associated with the pre-weaning survivability of Kalahari Red kids, although a positive trend was identified where heavier kids exhibited higher survival rates with kids born under 2 kg having a pre-weaning survivability of 93% and those with a birth weight greater than 6 kg having a 100% having a pre-weaning survivability. In conclusion, this study suggests that litter sizes 1–4 make for the most viable pre-weaning survivability rates and that, although not significantly associated in the current study, higher birth weights are positively associated with better pre-weaning survivability rates. The results of the current study will inform selection efforts and guide farmers’ breeding objectives to selecting for better litter size and birth weights whilst maintaining economically viable pre-weaning survivability rates.

## Introduction

Goats are crucial for agricultural production systems as they can utilise less water, are adaptive and can be productive in ecosystems that are harsh and marginal and can consume forage that is otherwise unpalatable to other agricultural species whilst producing highly nutritive products like milk and chevon amongst others (Mahmoudi et al. 2022). In South Africa, for the production of chevon, three main breeds are utilized including the Boer, Savannah and Kalahari Red goat breeds (Visser 2019). The Kalahari Red goat is one that is characterized by its adaptation to the arid and semi-arid savannah, by its light to dark-red coat colour, and its productive potential in the production of meat and has been described as having good mothering abilities (Snyman 2014; Visser 2018; Oderinwale et al. 2020).

Of the major limitations that are known to impact goat productions, pre-weaning mortality remains a major and crucial challenge, more so under extensive and smallholder farming systems (Deribe et al. 2014; Robertson et al. 2020; Datt et al. 2023). In the life of a goat kid, the period from birth to weaning is the most vulnerable stage as the kid is more susceptible to stress and their concurrent survival is influenced by genetic environmental maternal and management factors (Flowers 2015; Ataç 2022; Datt et al. 2023). Among the elements that can impact the survival of kids is the litter size and birth weight which directly affects neonatal viability and post-natal adaptation (Baiden 2007; Tesema et al. 2017).

The litter size and birth weights have been shown to have a dynamic and significant relationship with survival (Sodiq 2012). Lower individual birth weight and the greatest susceptibility to mortality are often because of high litter sizes, as this causes intrauterine competition for nutrients (Snyman 2010; Habtegiorgis et al. 2022). Similarly, low birth weight is a well-established risk factor for reduced thermoregulatory capacity, poor colostrum intake, and weakened immune response, thereby increasing mortality risk (El-Raghi and Hashem 2022; Datt et al. 2023). The mothering ability of the bred along with milk production capabilities can expound in this (Singh et al.^2022^). These challenges are further compounded under extensive management systems where nutritional supplementation and intensive neonatal care are often lacking (Deribe et al. 2014; Kumar et al. 2021). The Kalahari red goat breed is one that is valued for its adaptiveness, hardiness and productive efficiency even under semi-arid to arid conditions, however like other goat breeds their productivity can be hindered by early kid losses (Mokoena et al. 2022; Chen et al. 2024). Extensive information is available for reproductive performance in sheep breeds and limited in that of goats (Robertson et al. 2020). As such, understanding how the litter size and birth weight have an influence survivability in this breed is essential for designing management and selection strategies that are effective and sustainable.

The current study aimed to assess the effect of the litter size and the birth weight on the pre-weaning survivability of Kalahari Red goat kids. This study will be helpful to Kalahari Red goat breeders when developing a breeding strategy that will accommodate good litter size, birth weight whilst maintaining viable survival rates.

## Materials and methods

### Ethical clearance

The study was granted ethical clearance by the University of South Africa’s College of Agriculture and Environmental Sciences Animal Research Ethics Committee (Ethical reference number: 2024/CAES_AREC/6186).

### Data source

Animal records spanning 15 years (2009–2024) were retrieved from the South African Boer Goat Association through the South African Studbook. The data collected included the litter size, birth weight, and survivability. A dataset of 2390 animal records was retrieved for the purpose of analysis. The litter sizes analysed was from 1 to 5 and the birth weight was grouped into <2 kg, 2 –4 kg, 4 –6 kg and > 6 kg.

### Animal management

Animal that are part of the South African Studbook’s SA Boer Goat Association were selected to be part of the study. Animals were put into extensive production systems, where they are permitted to graze and forage from morning through to evening, with occasional supplementation, and are put into an enclosure at night with regular dipping protocol taking place against external parasites. The weaning of kids occurs on average at 90 days.

### Statistical analysis

Data were analysed using IBM SPSS Statistics (version 30.0, 2024). A two-way analysis of variance (ANOVA) using General Linear Model (GLM) procedure was used to evaluate the effects of litter size and birth weight on pre-weaning survivability. Birth weight was categorised into four categories (<2 kg, 2 –4 kg, 4 –6 kg, > 6 kg) and the litter size were from 1 to 5 kids. The interaction between litter size and birth weight was also investigated.

The following model was used:

$$Y_{ijk} = \mu +BW_{i} + LS_{j} + (BW x LS)_{ij} + e_{ijk}$$

Where:

Y_ijk_ = pre-weaning survivability, µ = overall mean, BW_i_ = effect of birth weight, LS_j_ = effect of litter size, (BW x LS) ij = the interaction between birth weight and litter size and e_ijk_ = random error term associated with each observation. Duncan Multiple Range Test was used to test the difference between means for different factors at *P* < 0.05.

## Results

### The effect of litter size on the pre-weaning survivability

The effect of litter size on the pre-weaning survivability in the Kalahari Red goats is detailed in Table 1. The results of the effect of the litter size on the pre-weaning survivability were shown to be statistically significant (*P* < 0.05). Litter sizes 1 to 4 differed significantly from litter size 5. Litter size was shown to be inversely proportional to the pre-weaning survivability.

**Table 1:** The effect of litter size on the pre-weaning survivability

Litter size	*N*	Pre-weaning survivability (%) ± SE
1	320	95 ± 1.20^a^
2	1367	94 ± 0.60^a^
3	633	91 ± 1.10^a^
4	57	89 ± 4.10^a^
5	13	69 ± 13.30^b^
**Total**	**2390**	**93 ± 0.05**

N: Sample size. SE: Standard error, Different superscript on the same column shows significant difference (P < 0.05)

### The effect of birth weight on the pre-weaning survivability

Weight at birth’s effect on the pre-weaning survivability in the Kalahari Red goats is detailed in Table 2. The results of the effect of the birth weight were shown to not have a significant effect on the pre-weaning survivability of Kalahari Red goat kids at *P* > 0.05.

**Table 2:** The effect of birth weight on the pre-weaning survivability

Birth Weight	*N*	Pre-weaning survivability (%) ± SE
<2 kg	203	93 ± 1.80
2–4 kg	1630	94 ± 0.60
4–6 kg	553	92 ± 1.20
> 6 kg	4	100 ± 0.00
**Total**	**2390**	**93 ± 0.50**

N: Sample size. SE: Standard error, Different superscript on the same column shows significant difference (P < 0.05)

## The effect of birth weight and litter size interaction on the pre-weaning survivability

The interaction of the birth weight and the litter size on the pre-weaning survivability in Kalahari Red goats is detailed in Table 3. The results on the birth weight x litter size were observed to not have a significant effect on the pre-weaning survivability of Kalahari red goats at *P* > 0.05.

**Table 3:** The effect of birth weight and litter size interaction on the pre-weaning survivability

Litter size	Birth weight	Pre-weaning survivability (%) ± SE
1	<2 kg	96 ± 0.05
	2–4 kg	98 ± 0.02
	4–6 kg	91 ± 0.02
	> 6 kg	100 ± 0.18
2	<2 kg	95 ± 0.02
	2–4 kg	95 ±0.01
	4–6 kg	92 ± 0.01
	> 6 kg	100 ± 0.18
3	<2 kg	87 ± 0.04
	2–4 kg	92 ± 0.01
	4–6 kg	92 ± 0.03
4	<2 kg	100 ± 0.14
	2–4 kg	89 ± 0.04
	4–6 kg	83 ± 0.10
5	<2 kg	50 ± 0.12
	2–4 kg	78 ± 0.10

## Discussion

Understanding the dynamic that occurs between reproductive traits like the litter size and birth weight of kids and their mortality is important as this can impact the viability of animal production processes and the enterprise (Khani et al. 2017). The effects of litter size and birth weight on the pre-weaning survivability of Kalahari Red kids were assessed in the current study. The results showed that litter size affected pre-weaning survivability, whereas birth weight had no significant effect.

The highest pre-weaning survival rates were shown in kid born into smaller litter sizes, whilst an increase in litter size decreased pre-weaning survivability in Kalahari red kids, with singletons and quintuplets showing a stark difference at 95% and 69% pre-weaning survivability respectively. The trend observed in the current study aligns with the findings by Muthukumar et al. (2016) in Tellichery goats, Tesema et al. (2017) in Central Highland x Boer crossbred goats, El-Raghi and Hashem (2022) in Zaraibi goats and Ataç (2022) in Turkish Saanen goats, who in their entirety reported that pre-weaning survival rates decrease with an increase in the litter size a doe has. This is further supported by Tesema et al. (2018) who in the Boer and Central Highland goat crossbreds observed that increased litter sizes negatively impacted kid’s survival. The decrease in pre-weaning survival rates may be because the occurrence of large litters may overwhelm the ability of the doe to provide its kids with the adequate amount of nutritional resrources, further increasing the risk of early deaths in the kids (Robertson et al. 2020). Moreover, the occurrence of multiple births is usually associated with intrauterine crowding which has the potential to reduce foetal development concurrently lowering the birth weight as observed in the study by Muthukumar et al. (2016).

The birth weight’s effect on the pre-weaning survivability of Kalahari Red kids albeit was not observed to be statistically significant in the current study was shown to have a positive relationship with the pre-weaning survivability, where an increase in birth weight favoured higher pre-weaning survival rates. Kids that belong to birth weight category greater than six (> 6 kg) showed a greater survival rates at 100% in comparison to the 92–94% in the birth weight ranges less that 6 kg in birth weights. Similar association was observed in the study by Rashidi et al. (2011) in the Markhoz goat breed where the birth weight and the pre-weaning survivability had a positive correlation in Markhoz goats. The studies by Muthukumar et al. (2016), Snyman (2010), and Ataç (2022) in the Tellicherry, Angora, Saanen breeds, respectively, further support the currently observed trend where favourable survival rates are observed in tandem with higher birth weights. The occurrence of these better pre-weaning survival rates may be due greater vigour, strong suckling reflexes, and better thermoregulatory ability (Baiden 2007; Muthukumar et al. 2016). Although the results of the current study aren’t significant, the biological significance of birth weight to the survivability of kids cannot be ignored as it has been confirmed that low-birth-weight kids are more prone to hypothermia, infection and starvation soon after birth, more so in extensive production systems (Husain et al. 1995; El-Raghi and Hashem 2022). In the current study the birth weight had a positive effect on the survivability although not significantly and this can be attributed to the Kalahari Red’s great mothering ability (Oderinwale et al. 2020).

The combined effect of litter size and birth weight is especially relevant to the survivability. Multiple studies have shown that twins and triplets tend to be lighter and more vulnerable than singletons (Singh et al. 1990; Chen et al. 2024). This interdependence suggests that both traits must be considered together when assessing survivability risks. The interaction between the birth weight and the litter sizes’ effect on the pre-weaning survivability of Kalahari Red kids did not interact significantly in the current study. Although this is the case, on average the observed patterns of the litter size and birth weight are consistent with their individual observations. The study by Jones et al. (2005) observed a significant interaction between the birth weight and litter size (twin status) an found that for a particular birth weight that singletons had a higher mortality rate than twins.

From a production perspective, the result of the current study indicates that to create a balance between productivity and survivability there will be a necessity for a management strategy that aims for a moderate prolificacy. Interventions like nutritional supplementation of pregnant and lactating does can improve the birth weight, viability of the kids, respectively. Such practices are supported by Tesema et al. (2018), who emphasized targeted feed supplementation to mitigate the physiological burdens associated with multiple births.

## Conclusion

The current study investigated the relationship between the birth weight and litter size to the pre-weaning survivability. Only litter size had a significant effect on the pre-weaning survivability of Kalahari Red goat kids. The results of this study showcase the importance of the individual effects and the interaction of the litter size and the birth weight. As is consistently demonstrated multiple births often result in smaller weighted kids, concurrently increasing the susceptibility to mortality. As such, the evaluation of these traits in tandem is essential when developing breeding strategies that are aimed at improving overall survivability in Kalahari Red kids.

## Data Availability

The data will be made available by the corresponding author on request.
